# Minimally invasive management of an ascending colonic perforation secondary to distal biliary stent migration: a multidisciplinary, novel laparoendoscopic approach

**DOI:** 10.1093/jscr/rjac404

**Published:** 2022-09-14

**Authors:** Karishma Kodia, Carlos T Huerta, Yingyot Arora, Carey Wickham, Amar R Deshpande, Nivedh Paluvoi

**Affiliations:** Division of Colon and Rectal Surgery, Department of Surgery, University of Miami Leonard Miller School of Medicine, Miami, FL, USA; Division of Colon and Rectal Surgery, Department of Surgery, University of Miami Leonard Miller School of Medicine, Miami, FL, USA; Division of Colon and Rectal Surgery, Department of Surgery, University of Miami Leonard Miller School of Medicine, Miami, FL, USA; Division of Colon and Rectal Surgery, Department of Surgery, University of Miami Leonard Miller School of Medicine, Miami, FL, USA; Division of Gastroenterology, Department of Medicine, University of Miami Leonard Miller School of Medicine, Miami, FL, USA; Division of Colon and Rectal Surgery, Department of Surgery, University of Miami Leonard Miller School of Medicine, Miami, FL, USA

## Abstract

Endobiliary stents placed for benign and malignant indications can spontaneously dislocate from the biliary system and migrate to the distal gastrointestinal tract. Stent migration can result in gastrointestinal perforation, with the most common locations in the sigmoid and distal colon, and may require surgical intervention. We describe the case of a 60-year-old female presenting with an ascending colonic perforation secondary to a dislodged plastic biliary stent placed for palliation of her gallbladder carcinoma. The patient was managed with a combined laparoendoscopic approach by a multidisciplinary team—gastroenterology performed an endoscopic stent retrieval and colorectal surgery identified the location of the perforation laparoscopically and performed colonic serosal repairs. The patient had an uneventful postoperative course and was discharged on postoperative day 4. This case demonstrates a novel minimally invasive laparoendoscopic approach at a high-volume academic center for the treatment of ascending colonic perforation secondary to biliary stent migration.

## INTRODUCTION

The use of biliary stents in the management of obstructive jaundice for both benign and malignant hepatobiliary and pancreatic pathologies is well described [1]. In 1980, Soehendra and Reynders-Fredenix first described the implementation of a transpapillary drain for palliative bile duct drainage, which ‘guarantees the physiological flow of bile into the duodenum’ [2]. Known complications include stent occlusion, cholangitis and stent migration [3, 4]. Migration of plastic stents is estimated to occur in about 10% of cases, while self-expandable metal stent migration is less than 1% comparatively [1, 5]. Risk factors for stent migration include common bile duct dilatation, complete sphincterotomy, balloon dilatation and stent insertion for more than one month [6]. Perforations that occur secondary to stent migration are commonly localized to the duodenum [7]. Although rare, distal stent migration may cause colonic perforation, which most frequently occurs in the sigmoid colon [8]. We present a case of a rare ascending colonic perforation secondary to plastic biliary stent migration managed by a hybrid laparoendoscopic approach to achieve stent retrieval and colonic repair.

## CASE PRESENTATION

**Figure 1:**
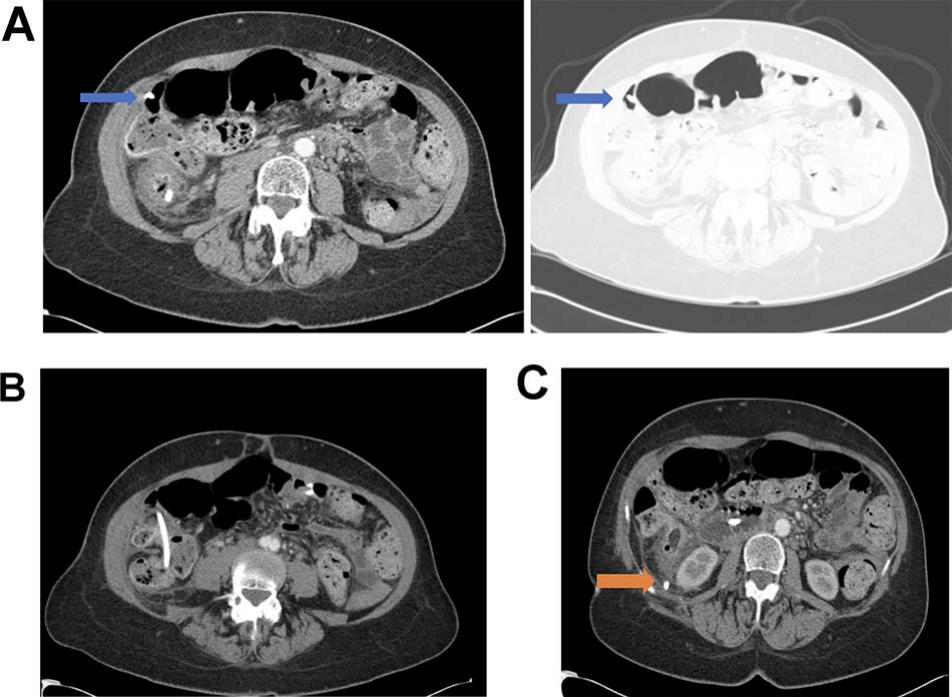
(**A**) Distal biliary stent perforation of the ascending colon at the level of the hepatic flexure (as indicated by arrows). (**B**) Stent migration at the level of the ascending colon. (**C**) Dislodged bile duct stent with perforation into the retroperitoneum at the level of the cecum (as indicated by arrow).

A 60-year-old female with a past medical history of metastatic gallbladder carcinoma presented to her primary oncologist with a 2-day history of abdominal pain. Her history was also significant for palliative biliary stents that had been placed 2 years ago and had been serially exchanged, most recently 2 months prior to presentation. Her oncologist obtained an outpatient abdominal computed tomography (CT) scan demonstrating the stent had caused a perforation of the right colon through the wall at the level of the hepatic flexure and perforation into the retroperitoneum at the level of the cecum without evidence of free intraabdominal air (Fig. 1A–C). The patient then presented to the emergency department for further evaluation. Upon evaluation, she was mildly tachycardic and normotensive with a temperature of 38.2°C. Physical examination was significant for mild abdominal distention and moderate tenderness to palpation in the right lower quadrant. Laboratory evaluation demonstrated a mild neutropenia but was otherwise within normal range. After extensive discussion with a multidisciplinary team including colorectal surgery, medical oncology, gastroenterology and the patient, a hybrid laparoendoscopic approach was planned to address stent removal versus a possible colectomy. The decision was made to start mechanical bowel preparation, despite possible perforation, to optimize an endoscopic attempt at retrieval, and the patient was taken to the operating room within 24 hours of presentation.

**Figure 2:**
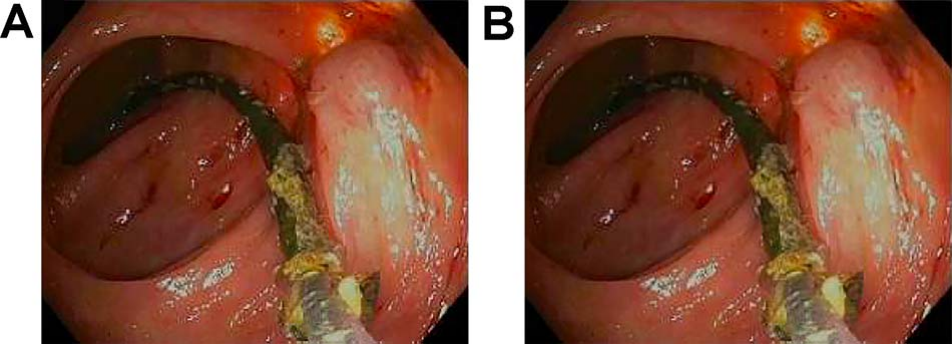
(**A**) Ascending colon demonstrating ulceration and the biliary stent lodged within colonic wall. (**B**) Stent across transverse colon with punctate erythema.

**Figure 3:**
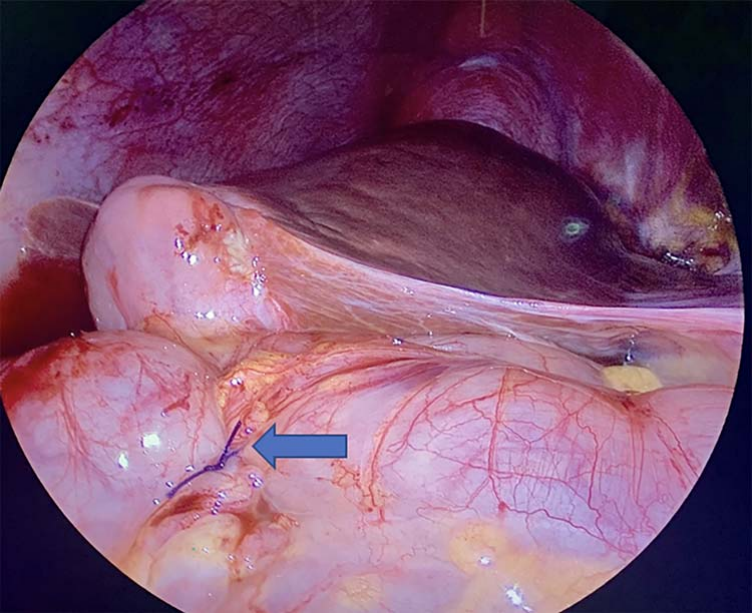
Laparoscopic visualization of a serosal lembert suture placement on transverse colon (as indicated by arrow; ascending colon serosal repair not featured).

Diagnostic laparoscopy of the abdomen was performed demonstrating diffuse carcinomatosis secondary to known primary gallbladder carcinoma. A portion of the ascending colon was adherent to the abdominal wall and was carefully dissected off. There was no full thickness erosion of the stent, and the stent was palpated through the colon at the ascending colon using bowel graspers. Colonoscopy was next performed, identifying the stent lodged in the mid-ascending colon and another intraluminal portion of the proximal transverse colon that appeared abnormal (Fig. 2A and B). The stent was successfully removed with rat tooth forceps by gastroenterology. The colorectal surgery team then performed two serosal suture repairs on the ascending and transverse colon (Fig. 3).

CT imaging obtained on postoperative day (POD) 3 was negative for any colonic pathology at which point she was initiated on a full liquid diet. The rest of her postoperative course was uneventful, and she was discharged on POD 4.

## DISCUSSION

Migration of biliary stents to the level of the colon is rare, and few case reports describe either isolated endoscopic or surgical management of such perforations [8]. Intra-abdominal risks of stent migration include fistula, abscess and perforation [1]. Depending on the location of the migrated stent, endoscopic options for management of stents that remain intraluminal include clip closure and retrieval of the stent using a helical basket, foreign body grasping forceps, snare forceps or stent cannulation techniques [6, 8]. Surgical options are indicated in cases of perforation causing peritonitis, abscess or fistulas. In one case, utilizing concomitant endoscopic and surgical approaches, Liang *et al*. described using a mini-laparotomy, upper endoscopy and fluoroscopy to address a biliary stent lodged in the jejunum that was ultimately milked proximally back to the level of the duodenum and removed by upper endoscopy [9]. In our case, given the intraluminal stent location and our laparoendoscopic approach, the patient was able to avoid a laparotomy and the associated morbidity, particularly important in the case of metastatic cancer with carcinomatosis. Laparoscopy allowed the patient to recover within days of her index case and remarkably she did not require any opioid pain medication postoperatively. In the current era of minimally invasive surgery (MIS) and endoscopy, select patients are served by ever-evolving MIS technology in the management of migrated stents.

Furthermore, Park *et al*., in a review of 30 cases between 1994 and 2021 of biliary stent migration to the colon, reported the majority of colonic perforations occurred in the sigmoid colon, with only one case of perforation each in the ascending colon and cecum [10]. This case of ascending colon perforation adds to the existing literature and reinforces that a high index of suspicion for perforation with stent migration should be considered for all segments of the small bowel and colon.

Early diagnosis in this case allowed the colorectal and gastroenterology teams to coalesce in a timely fashion to proceed aggressively to address the dislodged stent. As a tertiary referral center, the combined expertise of medical oncology, colorectal surgery and gastroenterology allowed for the patient to be expeditiously managed in a minimally invasive fashion, foregoing the need for an open exploratory laparotomy in an abdomen with metastatic disease and carcinomatosis. Ultimately, this management approach allowed the patient to return home in an expedient manner, important given her poor overall prognosis. To best of our knowledge, this case represents the first described hybrid laparoscopic and endoscopic approach with colorectal surgery and gastroenterology performing a combined case to remove a migrated biliary stent lodged in the ascending colon. In the current era, efforts should be made in appropriately selected patients to address stent migration in a minimally invasive fashion with a multidisciplinary group of gastroenterology and colorectal surgical teams.

## CONFLICT OF INTEREST STATEMENT

None declared.

## FUNDING

None.
